# Japanese encephalitis virus live attenuated vaccine strains display altered immunogenicity, virulence and genetic diversity

**DOI:** 10.1038/s41541-021-00371-y

**Published:** 2021-09-02

**Authors:** Emily H. Davis, Andrew S. Beck, Li Li, Mellodee M. White, Marianne Banks Greenberg, Jill K. Thompson, Steven G. Widen, Alan D. T. Barrett, Nigel Bourne

**Affiliations:** 1 Department of Pathology, The University of Texas Medical Branch, Galveston, TX USA; 2 Department of Pediatrics, The University of Texas Medical Branch, Galveston, TX USA; 3 Department of Biochemistry and Molecular Biology, The University of Texas Medical Branch, Galveston, TX USA; 4 Sealy Institute for Vaccine Sciences, The University of Texas Medical Branch, Galveston, TX USA; 5 Present Address: Viral Vaccine Preventable Diseases Branch, Division of Viral Diseases, Centers for Disease Control and Prevention, Atlanta, GA USA

**Keywords:** Live attenuated vaccines

## Abstract

Japanese encephalitis virus (JEV) is the etiological agent of Japanese encephalitis (JE). The most commonly used vaccine used to prevent JE is the live-attenuated strain SA14-14-2, which was generated by serial passage of the wild-type (WT) JEV strain SA14. Two other vaccine candidates, SA14-5-3 and SA14-2-8 were derived from SA14. Both were shown to be attenuated but lacked sufficient immunogenicity to be considered effective vaccines. To better contrast the SA14-14-2 vaccine with its less-immunogenic counterparts, genetic diversity, ribavirin sensitivity, mouse virulence and mouse immunogenicity of the three vaccines were investigated. Next generation sequencing demonstrated that SA14-14-2 was significantly more diverse than both SA14-5-3 and SA14-2-8, and was slightly less diverse than WT SA14. Notably, WT SA14 had unpredictable levels of diversity across its genome whereas SA14-14-2 is highly diverse, but genetic diversity is not random, rather the virus only tolerates variability at certain residues. Using Ribavirin sensitivity in vitro, it was found that SA14-14-2 has a lower fidelity replication complex compared to SA14-5-3 and SA14-2-8. Mouse virulence studies showed that SA14-2-8 was the most virulent of the three vaccine strains while SA14-14-2 had the most favorable combination of safety (virulence) and immunogenicity for all vaccines tested. SA14-14-2 contains genetic diversity and sensitivity to the antiviral Ribavirin similar to WT parent SA14, and this genetic diversity likely explains the (1) differences in genomic sequences reported for SA14-14-2 and (2) the encoding of major attenuation determinants by the viral E protein.

## Introduction

Japanese encephalitis virus (JEV) is the prototype member of the JE serogroup within the genus *Flavivirus*, which contains other mosquito-borne encephalitic viruses such as West Nile and St. Louis encephalitis. JEV is the leading cause of epidemic, viral encephalitis in mainland Asia and the Western Pacific causing an estimated 57,000 to 175,000 cases annually with a 20–30% case fatality rate. Thirty to fifty percent of those who survive the acute infection report serious neurological sequelae^1,2^. There are no licensed antivirals to treat the disease making vaccination the primary control strategy. A number of JEV vaccines have been licensed including live attenuated (LAV), chimeric LAVs and inactivated vaccines. The most commonly used in Asia is the LAV strain SA14-14-2, which is highly efficacious showing 90% seroconversion after a single immunization in multiple clinical trials^3–5^.

Strain SA14-14-2 was derived from the wild-type (WT) strain SA14 by plaque purification after serial passage in primary hamster kidney (PHK) cells and suckling mice^6^. The genomic sequences of SA14-14-2 and SA14 have been published a number of times but the published sequences differ between groups, which makes determination of attenuating mutations difficult^7–12^. While one study reports that there are no genomic changes after passage of SA14-14-2 in cell culture, mice or mosquitoes^13^ others report consensus changes after very few passages in similar substrates. These changes include three amino acid substitutions after passage in C6/36 cells, four amino acid substitutions after 22 passages in PHK cells, one amino acid substitution after passage in baby hamster kidney (BHK) cells, one amino acid substitution after passage in Vero cells, and four amino acid substitutions after passage in mouse brain^10,14,15^. There have been limited studies to resolve the differences in genomic sequences^12,16^ or the conditions that favor changes to the vaccine genotype^17^.

**Fig. 1:**
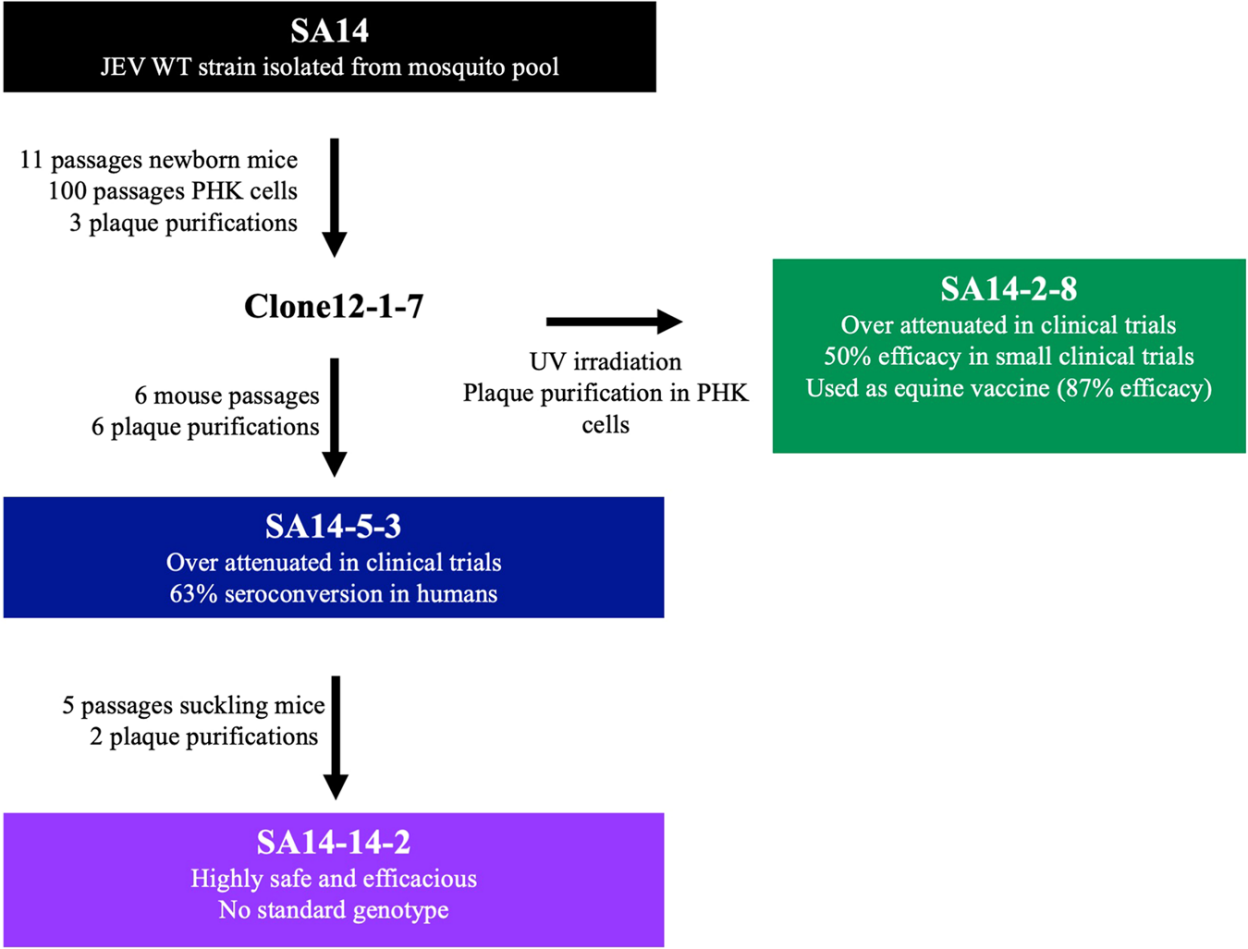
Derivation of JEV vaccine strains. The successful SA14-14-2 vaccine strain was derived after multiple attempts to attenuate WT strain SA14. The first of these attempts, Clone12-1-7, was generated by mouse and cell culture passage. It was then UV irradiated to generate SA14-2-8 and further mouse passaged to generate SA14-5-3. Both of these vaccine strains were shown to be overly attenuated in humans so SA14-5-3 was passaged in suckling mice which generated the currently utilized SA14-14-2.

There were multiple attempts to develop LAVs from SA14 prior to the generation of SA14-14-2 (see Fig. 1). Clone 12-1-7 was derived by plaque purification after passage in newborn mice and PHK cells. It was then UV irradiated, and plaque purified in PHK cells to produce strain SA14-2-8^18,19^. However, SA14-2-8 was poorly immunogenic in humans, producing seroconversion in only 50% of individuals, and was therefore used exclusively as an equine vaccine^8,18,20,21^. Strain SA14-5-3 was also derived from Clone12-1-7 following passage in mice and PHK cells but it was also over-attenuated with low immunogenicity in clinical trials^22,23^. Subsequently, SA14-5-3 was passaged in suckling mice and plaque purified, to derive SA14-14-2, which demonstrates enhanced immunogenicity while retaining an attenuated phenotype^6,13,23,24^.

JEV strains fall into a single serogroup that contains five genotypes. All current JEV vaccines are derived from genotype III. Licensed JEV vaccines appear to provide protective immunity against infection by the different genotypes, although the situation for genotype V is unclear^25–27^. To date there are only three isolates of genotype V and there are limited cross-neutralization data in vitro. Mouse studies have shown that sera from mice immunized with SA14-5-3 and SA14-2-8 exhibit limited cross neutralization against multiple genotypes whereas SA14-14-2 does provide effective cross neutralization^24,27–29^.

The flavivirus genome consists of three structural (capsid [C], premembrane [prM] and envelope [E]) and seven non-structural (NS) genes (NS1, NS2A, NS2B, NS3, NS4A, NS4B and NS5). The E protein is the major antigenic driver of the humoral immune response to the virus, thereby dictating the success of vaccine cross-protection. Studies have implicated substitutions in the E protein as the major determinants of attenuation of SA14-14-2^9,13,15,17,30–38^ with the NS proteins, which constitute the replication complex, playing no or a very limited role^11,17,39,40^. In fact, a chimeric SA14 virus containing SA14-14-2 prM/E genes was completely attenuated in a 3-week-old Kumming mouse model, demonstrating the importance of E protein in attenuation^41^. This mechanism of attenuation differs from that of another flavivirus LAV, yellow fever (YF) 17D where both structural and NS proteins contribute to the attenuated phenotype. It has been shown that the genome of the 17D vaccine virus is significantly less diverse than the WT parent strain from which it was derived^42,43^. The homogenous genotype of 17D has been attributed to the increased fidelity of the replication complex, which was shown using resistance to the nucleoside analog Ribavirin and is thought to contribute to the stability and safety of the vaccine^44^. Next generation sequencing (NGS) technology has yet to be applied to JEV in these contexts and so the diversity of the WT SA14 and vaccine strains is unknown. The variability of the SA14-14-2 genomic sequences described above suggests it displays more diversity than YFV 17D, potentially distinguishing their mechanisms of attenuation despite similar empirical derivation strategies and clinical efficacies.

WT JEV infection has been modeled using adult and suckling mice, and multiple outbred and inbred strains^10,11,13,17,31,34,45–49^. In general, other than newborn mice, WT JEV strains only cause a lethal infection when given intracranially or by a peripheral route after the blood brain barrier has been perturbed whereas it has been shown that SA14-14-2 is avirulent in suckling mice by both intracranial and intraperitoneal routes (Yang, 2014). JEV LAVs are 100% attenuated in most of these models and in many cases are cleared so efficiently that no neutralizing antibody response is generated. For SA14-14-2, a dose of one million pfu is non-lethal in immunocompetent mice even if the virus is administered directly into the brain^36^. SA14-14-2 has only shown virulence in interferon-αβγ receptor knockout (AG129) mice^47^.

In order to better understand the attenuated phenotype of SA14-14-2, we have undertaken a series of studies that compare genetic diversity, mouse virulence phenotype and immunogenicity of the vaccine strains derived from WT strain SA14 (SA14-14-2, SA14-5-3 and SA14-2-8). We demonstrate that SA14-14-2 has both high genetic diversity and sensitivity to the antiviral Ribavirin similar to WT parent SA14. This apparent genetic diversity likely explains the differences in genomic sequences reported for SA14-14-2 plus why the E protein encodes the major determinants of attenuation. In addition, despite displaying a mechanism of attenuation that distinguishes it from YF 17D LAV, SA14-14-2 maintains a similar level of attenuation in susceptible mouse models.

## Results

### Consensus genomic sequences of WT SA14 and JEV vaccine viruses used in studies

The consensus sequences of virus stocks used in the studies were determined by NGS and compared to published sequences of SA14 (GenBank references KU323483, U14163, KU821122, MH258848, KX254415, KU871316)^10,12^ (Table S1), SA14-14-2 (GenBank references: MH258849,MH258850, MH258851, MH258852, MH258853, KX254416, KX254417, D90195, MK585066, AF315119 and JN604986)^10,50–52^ (Table 1), SA14-2-8 (Genbank reference: U15763)^12^ (Table S2) and SA14-5-3 (Genbank reference: U04521) **(**Table S3**)**. Only a partial sequence has been published for SA14-5-3.

**Table 1:** Comparison of SA14-14-2 in this study and twelve previously genomic sequences from Genbank.

GenBank Accession number						AF416457	MH258851	MH258849	MH258853	MH258852	MH258850	KX254416	KX254417	D90195	MK585066	AF315119	JN604986
Nucleotide Position	CDS Position	Polyprotein	Gene	AA protein #	SA14-14-2	SA14-2-1-7	SA14-14-2	SA14-14-2	SA14-14-2	SA14-14-2	SA14-14-2	SA14-14-2	SA14-14-2	SA14-14-2	SA14-14-2	SA14-14-2	SA14-14-2
21	−74	−24	5′UTR	—	—	—											
39	−56	−18	5′UTR	—	—												
59	−36	−12	5′UTR	—	—						—				-		
127	32	11	C	11	N	S											
292	197	66	C	66	S												
316	221	74	C	74	K	R											
375	280	94	C	94	A	T											
518	423	141	prM	14	I										*		
884	789	263	prM	136	F										*		
1017	922	308	E	14	G	R											
1061	966	322	E	28	D												
1086	991	331	E	37	D										N		
1117	1022	341	E	47	S	N	N	N	N	N	N	N	N	N	N	N	N
1217	1122	374	E	80	A												
1296	1201	401	E	107	F												
1389	1294	432	E	138	K												
1503	1408	470	E	176	V												
1506	1411	471	E	177	A	T											
1512	1417	473	E	179	E	K	K	K	K	K	K	K	K	K	K	K	K
1532	1437	479	E	185	E	*											
1769	1674	558	E	264	H												
1813	1718	573	E	279	K		M	M	M	M	M			M	M	M	
1921	1826	609	E	315	V	A											
1977	1882	628	E	334	P												
2012	1917	639	E	345	L	*											
2143	2048	683	E	389	D										A		
2293	2198	733	E	439	R												
2317	2222	741	E	447	G											D	
2441	2346	782	E	488	G	*											
2691	2596	866	NS1	72	R												
2843	2748	916	NS1	122	I												
3351	3256	1086	NS1	292	S	G											
3493	3398	1133	NS1	339	M	R											
3516	3421	1141	NS1	347	R												
3528	3433	1145	NS1	351	H	D											
3530	3435	1145	NS1	351	H	*											
3539	3444	1148	NS1	354	K												
3599	3504	1168	NS1	374	E												
3652	3557	1186	NS1	392	V	A											
3677	3582	1194	NS1	400	G												
3776	3681	1227	NS2A	18	A												
3801	3706	1236	NS2A	27	L												
3929	3834	1278	NS2A	69	A									*			
4106	4011	1337	NS2A	128	A	*											
4250	4155	1385	NS2B	12	G	*											
4403	4308	1436	NS2B	63	D	E											
4408	4313	1438	NS2B	65	G												
4475	4380	1460	NS2B	87	L											F	
4782	4687	1563	NS3	59	V												
4825	4730	1577	NS3	73	K												
4862	4767	1589	NS3	85	F										*		
4921	4826	1609	NS3	105	G												
4922	4827	1609	NS3	105	G												
5230	5135	1712	NS3	208	I	T											
5234	5139	1713	NS3	209	I												
5311	5216	1739	NS3	235	V	A											A
5634	5539	1847	NS3	343	R									W			
5994	5899	1967	NS3	463	G	S											
6008	5913	1971	NS3	467	N												
6035	5940	1980	NS3	476	G										*		
6425	6330	2110	NS3	606	Q												
6634	6539	2180	NS4A	57	I									T			
6728	6633	2211	NS4A	88	T	*											
6904	6809	2270	NS4A	147	V	A											
6944	6849	2283	NS4A	160	A	*											
7005	6910	2304	NS4A	181	M	V											
7121	7026	2342	NS4A	219	A												
7193	7098	2366	NS4A	243	T												
7227	7132	2378	NS4A	255	V												
7295	7200	2400	NS4B	10	G	*	*	*	*	*	*			*	*	*	*
7655	7560	2520	NS4B	130	A									*			
7656	7561	2521	NS4B	131	N	D	D	D	D	D	D	D	D	D	D	D	D
7736	7641	2547	NS5	20	S	*											
7768	7673	2558	NS5	31	A											G	
7809	7714	2572	NS5	45	R											S	
7871	7776	2592	NS5	65	L											*	
7926	7831	2611	NS5	84	R	C											
8067	7972	2658	NS5	131	D	*											
8099	8004	2668	NS5	141	D												
8261	8166	2722	NS5	195	M											I	
8276	8181	2727	NS5	200	R	*										*	
8394	8299	2767	NS5	240	L												
8832	8737	2913	NS5	386	Y	H											
8882	8787	2929	NS5	402	I	*											
8891	8796	2932	NS5	405	V												
9122	9027	3009	NS5	482	G	*	*	*	*	*	*	*	*	*	*	*	*
9593	9498	3166	NS5	639	H	Q								Q			
9688	9593	3198	NS5	671	A	V											
9695	9600	3200	NS5	673	K												
9818	9723	3241	NS5	714	C												
9898	9803	3268	NS5	741	G	D											
9917	9822	3274	NS5	747	P												*
9954	9859	3287	NS5	760	A											P	
9971	9876	3292	NS5	765	Q											*	
9978	9883	3295	NS5	768	L											V	
9995	9900	3300	NS5	773	H											*	
10046	9951	3317	NS5	790	V												
10139	10044	3348	NS5	821	V												
10217	10122	3374	NS5	847	R												
10428	10333	3445	3’UTR	918	—	—											
10551	10456	3486	3’UTR	959	—	—											
10574	10479	3493	3’UTR	966	—											—	

Nucleotide position, coding sequence (CDS) nucleotide positions, polyprotein amino acid (AA) position, gene and amino acid position within protein are recorded. Nucleotide changes that do not occur within the CDS and therefore do not result in an amino acid change are recorded as (—). Nucleotide changes that are within the CDS but do not result in an amino acid change are recorded as (*). Single letter amino acid residue codes are used below.

The WT strain SA14 used in our studies was found to have three to eleven nucleotide changes encoding two to six amino acid substitutions compared to previously published SA14 strain sequences^10,12^. None of these changes were conserved across GenBank submissions though one change, 1708 (E-G244E), was reported in five of six publications.

The SA14-14-2 stock used in our studies differed from all previously published isolates by four nucleotides that encoded one synonymous change at nucleotide 9122 and three non-synonymous consensus changes at nucleotides 1117 (E-N47S), 1512 (E-K179E) and 7656 (NS4B-D131N)^10,51,52^ (Table 1). As residues E-47N, E-179K and NS4B-131D were present in all previously published SA14-14-2 strains and in progenitor Clone12-1-7 (Genbank submission: AF416457), it can be presumed that these residues constitute part of a conserved vaccine genotype. The changes detected in the SA14-14-2 strain utilized in these studies (E-47S, E-179E and NS4B-131N) would therefore be considered mutations from this genotype, though not necessarily reversions to WT. Compared to the only other previously published commercial vaccine, the vaccine ampule of SA14-14-2 used in this study differs by three residues (E-N47S, E-M279K, and NS4B-D131N, Table 1)^10^.

**Fig. 2:**
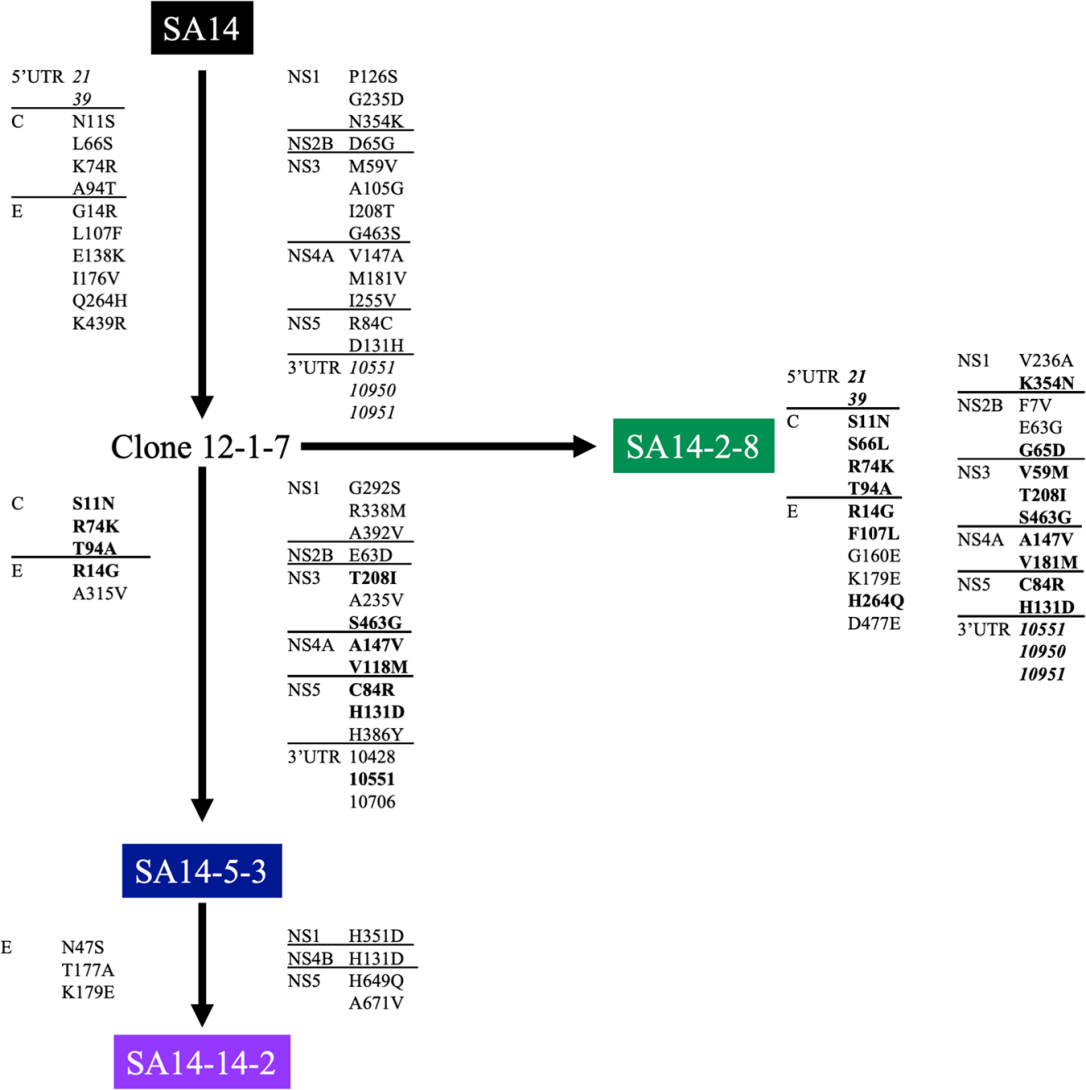
Consensus changes during JEV vaccine derivation. The complete genomes of SA14, Clone12-1-7, SA14-2-8, SA14-5-3 and SA14-14-2 were compared. Consensus changes that represent a reversion to the parental, WT strain SA14 are bolded. Consensus changes that occur within the protein coding region of the genome are reported as the codon number within the gene. Consensus changes that occur outside the protein coding region are italicized and reported as a nucleotide number. Sequences of SA14, SA14-2-8, SA14-5-3 and SA14-14-2 were generated in house through Illumina sequencing. The sequence for Clone-12-1-7 was accessed from GenBank (AF416457).

The SA14-5-3 stock used here differed from that published at three nucleotides that encoded two amino acid differences (5’UTR-C20G, prM-C34R and E-G244E)^12^. SA14-5-3 is highly similar to SA14-14-2, with only seven residues (E-N47S, E-T177A, E-K179E, NS1-H351D, NS4B-H131SD, NS5-H649Q, NS5-A671V) distinguishing the two viruses (Fig. 2) suggesting these residues contribute to the increased immunogenicity of SA14-14-2 compared to SA14-5-3 in clinical trials^22,23^.

The SA14-2-8 stock used in these studies differs from the one GenBank submission at 37 nucleotides, 13 of which result in non-synonymous amino acid (aa) mutations. Only one residue, E-315A, was determined to have a WT residue where the previously published sequence (E-315V) did not. Five WT residues (prM-88K, E-160G, E-477D, NS2B-7F and NS3-105V) were reported in the published sequence. Many of the residues in the SA14-2-8 strain used here that differ from the GenBank sequence are shared with SA14-14-2^16^. It is also apparent that SA14-2-8 is very similar to SA14 with only seven amino acid residues distinguishing the structural proteins of the two viruses (M-K88R, E-K138E, E-E160G, E-V176I, E-E179K, R439K and E-E477D) (Table S4), plus six residues in NS proteins (NS1-G235D, NS1-V236A, NS2B-F7V, NS2B-E63G, NS3-A105G and NS4A-I255V).

Overall, the WT progenitor SA14 differed from SA14-14-2 at 57 nucleotides encoding 25 aa, from SA14-5-3 at 47 nt and 21 aa and from SA14-2-8 at 35 nucleotides encoding 13 amino acids (Table S4). When compared to Clone 12-1-7, the attenuated progenitor of all vaccine strains, SA14 differs by 50 nt and 26 aa, SA14-14-2 differs by 41 nt and 24 aa, SA14-5-3 differs by 30 nt and 19 aa and SA14-2-8 differs by 52 nt and 28 aa. Common attenuating mutations in all three vaccines include: E-E138K, E-I176V, NS1-P126S, NS1-G235D, NS2B-E63D, NS3-A105G and NS4A-I255V.

### Consensus genome sequencing shows JEV vaccine viruses are stable upon passaging

We examined the impact of passaging on the stability of the consensus sequences of the WT and vaccine viruses used in these studies (Table S5). The SA14-14-2 vaccine stock was passaged once in C6/36 and twice in Vero cells resulting in one non-synonymous change at nt 1512 (E-K179E) and one synonymous change (NS5-G9122A) (Table S5A). SA14-2-8 incurred four changes, two synonymous (C-U158A and NS1-A3482U) and two non-synonymous nucleotide changes, 1447 and 4224 corresponding to residue changes E-G160E and NS2B-F7V), after one passage in Vero cells (Table S5B). Neither non-synonymous changes are reversion to WT SA14 nucleotides. No consensus changes to the SA14-5-3 genome were recorded upon passage in Vero cells.

### Next generation sequencing of JEV vaccine strains contrasts levels of diversity within the viral populations

**Fig. 3:**
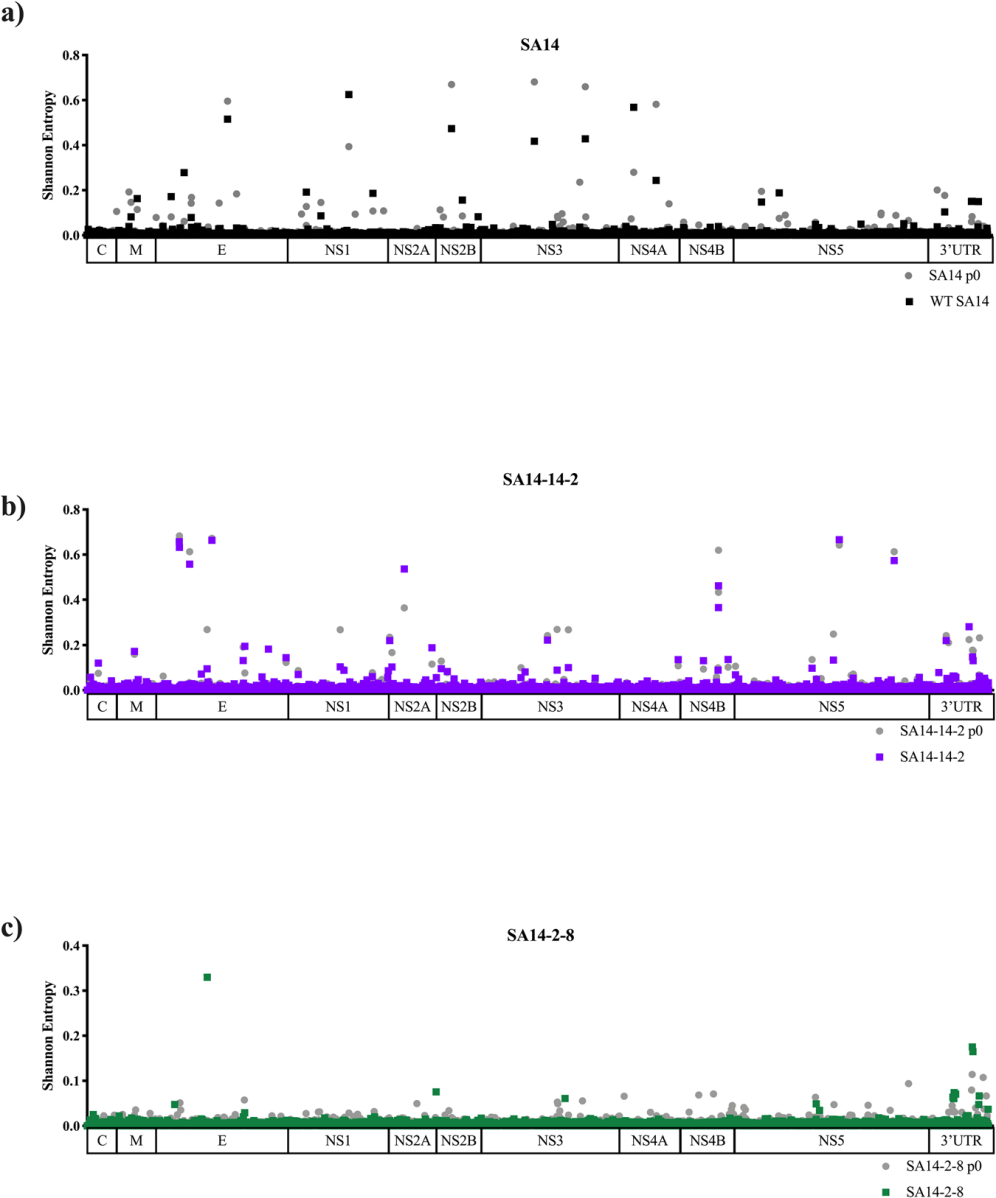
Genetic diversity of JEV WT and vaccine strains upon passage. The genetic diversity of two sequential passages of SA14 and the three vaccine strains derived from it was compared through Illumina sequencing methods and quantified using Shannon entropy. The Shannon entropy of SA14 (**a**), SA14-14-2 (**b**), SA14-5-3 (**c**) and SA14-2-8 (**d**) were mapped across the genome. All nucleotide positions, UTRs included, are depicted.

All strains used in these studies underwent Illumina sequencing and genetic diversity profiles were compared by calculation of Shannon’s entropy and identification of single nucleotide variants (SNVs)^53,54^. In addition to consensus sequence determination, the diversity of unpassaged (p0) SA14, SA14-14-2 and SA14-2-8 were determined and compared to the final passage of each virus (Fig. 3). The depth of coverage for each virus was as follows: SA14 p0 (4,360 reads/base) and p1 (10,009 reads/base), SA14-14-2 p0 (5,554 reads/base) and p1 (2,829 reads/base), SA14-2-8 p0 (3,155 reads/base) and P1(15,521 reads/base) and SA14-5-3 p0 (6,481 reads/base). Only p0 is presented for SA14-5-3 as p1 recorded low coverage levels and therefore only the consensus sequence could be determined from this sample. As the sample has the same consensus sequence as the previous passage, it is suggested that the virus has low diversity due its derivation and it is considered likely that p1 displays similar levels of diversity. Overall, SA14-2-8 and SA14-5-3 showed the least genetic diversity, WT SA14 the most genetic diversity, and SA14-14-2 was intermediate.

SA14 contained unpredictable levels of diversity across its genome, e.g., localizations of diversity were not conserved between passages. The SNV profile of the virus showed SNVs at locations that differentiate the SA14 used in this study from the SA14 strains published in GenBank. SNVs at residues that differentiate the previous reports occurred at prM-D21G (1.6%), E-G244E (21.02%), NS1-I122V (1.7%), NS1-G135D (31.5%), NS2B-S71S (18.01%), NS3-R275R (14.7%), NS3-D482N (15.4%) and NS4A-V58V (25.4%). SNVs were detected at the position of three synonymous mutations differentiating the published sequences and the SA14 used here (NS2B-71, NS3-275 and NS4A-58); however, the nucleotide detected as a SNV was not the same as the nucleotide in the published sequence. No SNVs were detected at E-107 or E-138, which have been shown to be virulence determinants of SA14^10,11^. This reinforces our hypothesis that the WT SA14 is naturally genotypically heterogeneous, leading to consensus sequences that vary from sample to sample due to the expansion of subpopulations of viral RNA species. Interestingly, one SNV recorded a residue change at NS1-I122V (1.6%) whereas the consensus nucleotide differences between published strains and the strain used here were synonymous^10,51,52^.

**Fig. 4:**
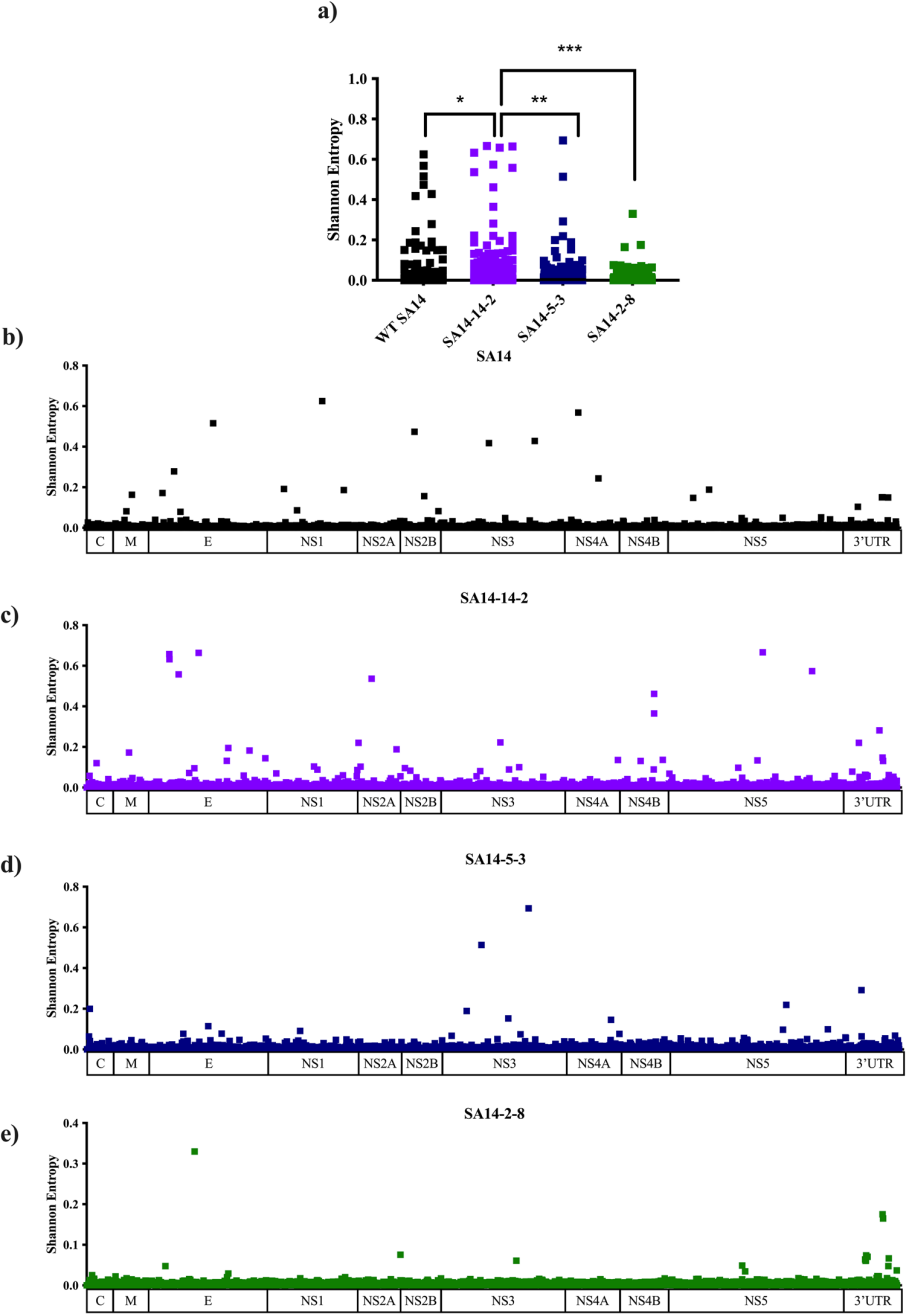
Genetic diversity of JEV vaccine strains compared to parental WT strain. The genetic diversity of SA14 and the three vaccine strains derived from it was compared through Illumina sequencing methods and quantified using Shannon entropy (**a**). The Shannon entropy of SA14 (**b**), SA14-14-2 (**c**), SA14-5-3 (**d**) and SA14-2-8 (**e**) were mapped across the genome. All nucleotide positions, UTRs included, are depicted in (**a**): *p = 0.014 (SA14 vs SA14-14-2), **p = 0.0003 (SA14-14-2 vs SA14-5-3), ***p < 0.0001 (SA14-14-2 vs SA14-2-8).

SA14-14-2 exhibits significantly lower levels of entropy compared to WT SA14 (*p* = 0.014) (Fig. 4a) and significantly higher levels of entropy than SA14-5-3 (*p* < 0.003) and SA14-2-8 (*p* < 0.0001). Areas of the genome displaying high diversity in WT SA14 did not overlap with those showing high diversity in SA14-14-2 suggesting diversity of SA14-14-2 was due to selection during passage in PHK cells and mice, rather than as a consequence of derivation from SA14 (Fig. 4b, c). For SA14-14-2, the E protein gene displayed the highest levels of diversity, followed by NS5 (Fig. 4b). Additionally, there were peaks of diversity at NS2A-41 and NS4B-104.

**Fig. 5:**
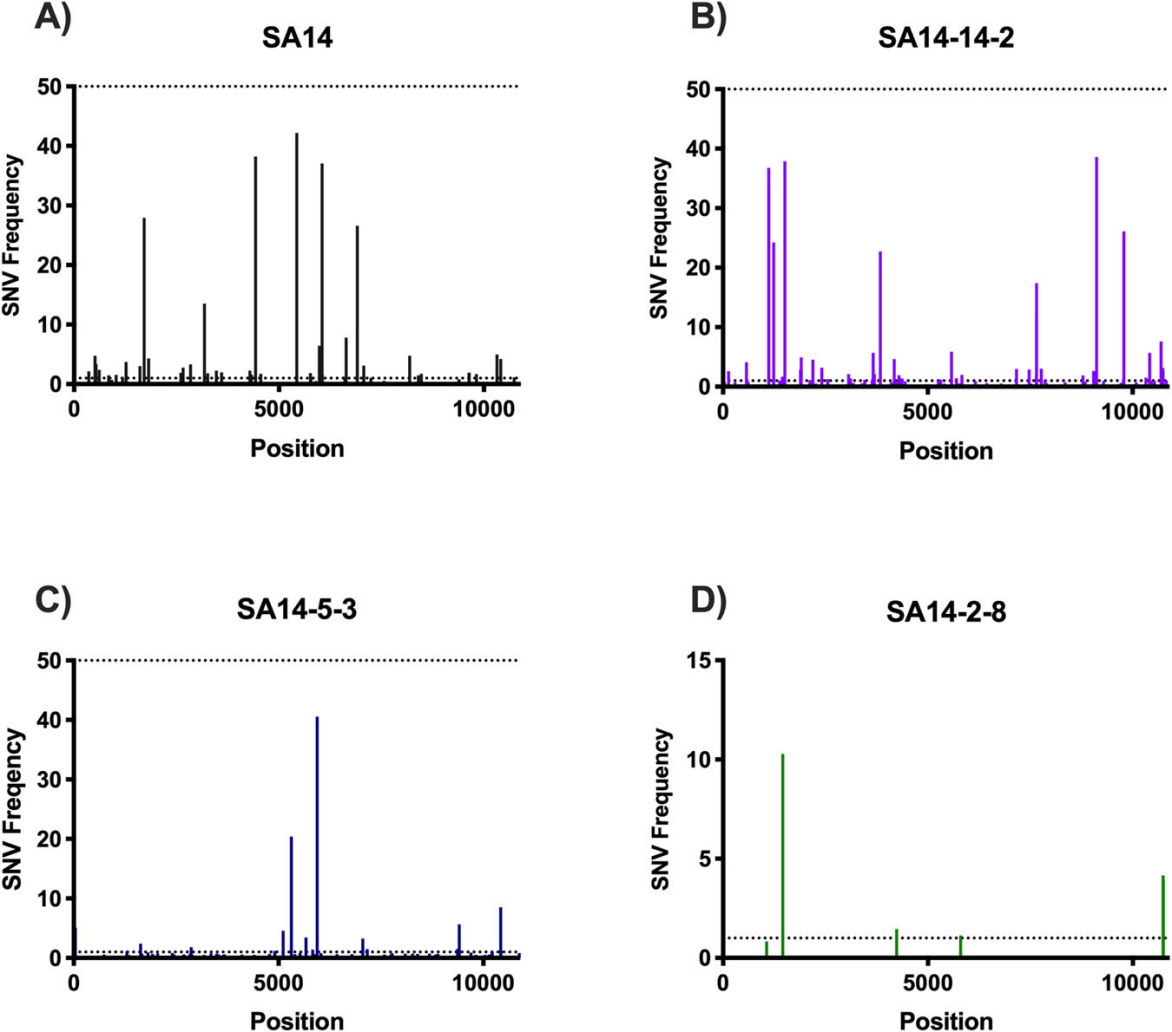
Frequency and genome location of single nucleotide variants detected in SA14 and vaccine derivatives. RNA was extracted and sequenced using Illumina methods. LoFreq software was used to detect SNVs in SA14 (**A**), SA14-14-2 (**B**), SA14-5-3 (**C**) and SA14-2-8 (**D**) samples. Dotted lines represent 50% of the population at which an SNV would become the consensus nucleotide and 1% of the population. An SNV frequency cutoff was applied at 0.05% frequency.

The differences between the diversity profiles of SA14 and SA14-14-2 are even more apparent when comparing SNV profiles (Fig. 5A, B, Tables S6 and S7). Both SA14 and SA14-14-2 contain SNVs that are near 50% of the population, or a consensus change. Three of these high frequency SNVs detected in SA14-14-2 (A1116G – 37%, G1117A – 33%, G9122A – 39%) overlap with SA14 identity. The changes at 1116 and 1117 correspond to minority residue changes at E-E47D. In the vaccine strain, the WT nucleotide is the minority variant upon final passage. When the SNV profile of SA14-14-2 is compared to previously published consensus sequences of SA14-14-2, SNVs are detected at residues E-S47N (37.01%), E-E179K (38.1%), NS1-V392G (1.3%), NS3-V235A (1.2%), NS4B-N131D (17.4%), and NS5-G482G (38.6%) that differentiate the SA14-14-2 used in this study from those published previously^10,51,52^. The nucleotide change (G9122A) associated with NS5-482 results in a synonymous change. Upon passaging of SA14-14-2, there was almost complete overlap in diversity peaks suggesting that even though SA14-14-2 is highly diverse, this genetic diversity is not random, rather the virus only tolerates variability at certain residues.

Very low levels of diversity were found across the entire genome of SA14-5-3 (Fig. 4d and Table S8). Only two peaks of entropy were recorded, both in NS3 (NS3-235 and NS3-446). These positions do not differentiate SA14-5-3 from SA14. Two high frequency, non-synonymous SNVs were also detected at these positions (NS3-V235A (3.5%) and NS3-N446K (40.5%)) but these SNVs did not lead to a consensus change upon passage.

SA14-2-8 displayed very low levels of entropy (Fig. 4d) and few, low frequency SNVs (Table S9). This was expected as the virus was derived by (1) UV irradiation and was then (2) plaque purified during its attenuation from SA14. There are peaks of entropy at E-160 and NS2B-7 that represent reversions to WT SA14. These few areas of heterogenicity are reproduced after passage and correspond with SNVs of frequency 10% and 1.5%, respectively. Overall, the average entropy and SNV profile was not changed during passage. Both initial and final passage of SA14-2-8 showed very low levels of diversity across the entire genome.

### SA14, SA14-14-2 and SA14-5-3 are susceptible to Ribavirin whereas SA14-2-8 is not

**Fig. 6:**
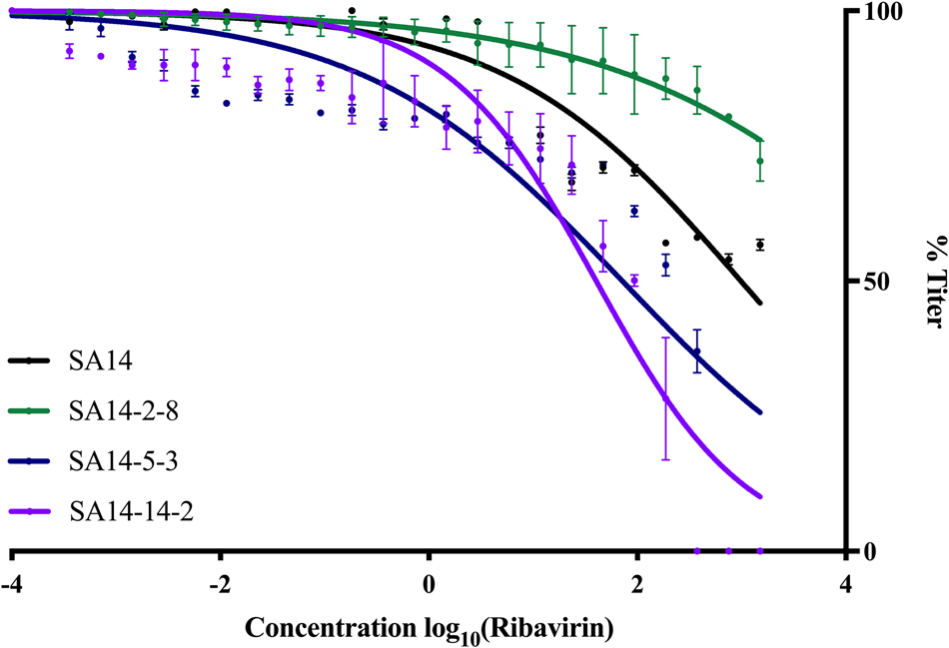
Dose response of WT JEV and JEV vaccine strains to the antiviral ribavirin. SA14, SA14-14-2, SA14-5-3 and SA14-2-8 were incubated with the GTP nucleoside analog, ribavirin. After 48 h, the supernatant was collected and titrated for viral load (FFU). Titers at each concentration were normalized to untreated, infected cells and fit using a dose-response linear regression. The experiment was undertaken in triplicate and points shown are an average of these experiments. A four-parameter, non-linear regression was used to fit sensitivity curves (SA14 *R*^2 ^= 0.89, SA14-14-2 *R*^2 ^= 0.89, SA14-5-3 *R*^2 ^= 0.79, SA14-2-8 *R*^2 ^= 0.964).

The susceptibility of WT JEV strain SA14 and the three vaccine strains derived from it to Ribavirin was determined in Vero cells. The titer reduction curve for SA14 was not significantly different than those for SA14-5-3 and SA14-2-8 (*p* = 0.087 and *p* = 0.389, respectively) but was significantly different from SA14-14-2 (*p* = 0.016) (Fig. 6). The SA14-14-2 viral reduction curve was significantly different from the SA14-2-8 curve (*p* < 0.0001) but not from the SA14-5-3 curve (*p* = 0.992) (Fig. 6). Titer reduction curves of SA14-5-3 and SA14-2-8 differed significantly from each other (*p* = 0.002) (Fig. 6). The 50% inhibitory concentrations (IC_50_) of SA14, SA14-14-2, SA14-5-3 and SA14-2-8 were 8.87 µM (*R*^2^ = 0.887), 0.40 µM (*R*^2 ^= 0.888), 0.68 µM (*R*^2 ^= 0.785) and 779.72 μM (*R*^2 ^= 0.964), respectively. This suggests that attenuation of SA14 to generate SA14-5-3 and SA14-14-2 by cell culture adaptation resulted in development of Ribavirin sensitivity (low-fidelity replication) while the derivation of SA14-2-8 by ultraviolet irradiation resulted in resistance (high-fidelity replication) to Ribavirin.

### SA14-14-2 and SA14-2-8 are highly virulent in interferon αβγ (AG129) receptor knockout mouse model whereas SA14-5-3 is attenuated

AG129 mice were inoculated with 1000 pfu of SA14-14-2, SA14-2-8 or SA14-5-3. Both SA14-14-2 and SA14-2-8 produced 100% lethality whereas neither lethality nor morbidity was observed with SA14-5-3. Mice infected with SA14-14-2 had a mean survival time (MST) of 10.0±0.3 days whereas the MST of mice infected with SA14-2-8 was 7.0 + 0.2 days (*p* < 0.0001; Table 2). In addition, viremia in SA14-2-8 infected mice were significantly higher at 3 dpi than for SA14-14-2 (*p* = 0.0002)(Table 2). In contrast, at the time of euthanasia, viral titers in the brain of SA14-2-8 infected mice were significantly lower than in SA14-14-2 animals (*p* = 0.015) (Table 2). Unsurprisingly, SA14-5-3 viremia at 3 dpi was significantly lower than for SA14-14-2 and SA14-2-8 (*p* < 0.001) (Table 2). At the end of the study, mice infected with SA14-5-3 were found to have high FRNT_50_ titers (mean reciprocal titer = 1274±162) indicating that in the highly susceptible AG129 mouse model, SA14-5-3 is both attenuated and immunogenic (Table 2).

**Table 2:** Outcome of subcutaneous infection of AG129 mice with JEV vaccine strains.

Mouse Model: 6-week old AG129					
Strain:	Number of mice	Mean survival time	Viremia (log_10_titer)	Mean PRNT_50_ (reciprocal)	Mean titer in brain (log_10_ titer)
SA14-14-2	16	10	7.3 ± 0.4	N/A	7.5 ± 0.6
SA14-2-8	16	7	8.1 ± 0.2	N/A	5.9 ± 0.6
SA14-5-3	18	N/A	5.8 ± 0.4	1274	N/A

### SA14-14-2 is more attenuated and more immunogenic than SA14-2-8 in the type-1 in interferon αβ receptor knockout (A129) mouse model

We also examined the virulence and immunogenicity of SA14-14-2 and SA14-2-8 in A129 mice, which lack only type-1 interferon signaling. As SA14-5-3 was fully attenuated in the more susceptible AG129 model, the virus was not assessed in A129 mice. Mice were again inoculated with 1000 pfu. SA14-14-2 was completely attenuated (100% survival) whereas SA14-2-8 had a mortality rate of 62.5% (Mean Survival Time [MST] of 7.0 ± 0.4dpi) (Table 3). Mice infected with SA14-14-2 and mice surviving SA14-2-8 infection had a strong neutralizing antibody response, though SA14-14-2 antibody titers were significantly higher than those induced by SA14-2-8 (*p* = 0.0135, mean FRNT_50_ titer: 3840± 624 and 2133± 427, respectively) (Table 3). Viremia in mice 3 dpi with SA14-2-8, was significantly higher (*p* = 0.0004) than in mice infected with SA14-14-2 (Table 3). The average viral infectivity titer in the brains of mice that succumbed to SA14-2-8 infection was 3.9x10^3^FFU/g at the time of death.

**Table 3:** Outcome of subcutaneous inoculation of A129 mice with JEV vaccine strains.

Mouse Model: 6-week old AG129					
Strain:	Number of mice	Mean survival time	Viremia (log_10_ titer)	Mean PRNT_50_ (reciprocal)	Mean titer in brain Log_10_ titer
SA14-14-2	7	N/A	7.0 ± 0.7	3840	N/A
SA14-2-8	8	7	8.0 ± 0.5	2133	3.6 ± 0.7

### Changes to the E protein were detected in SA14-14-2 but not SA14-2-8 when isolated from brains of AG129 mice

Virus recovered at euthanasia from the brains of mice infected with SA14-14-2 was sequenced using Illumina methods. In all brains consensus changes occurred at E-S47D and E-E179K. Both were SNVs detected in the sequencing studies described above suggesting that these residues are important to how SA14-14-2 enters or replicates in the brain of mice. E-47 differentiates SA14-14-2 and SA14-5-3 from SA14. The E-47 amino acid substitution detected in mouse brains (aspartic acid) is a reversion to SA14-5-3 not to WT SA14 (asparagine). No consensus residue changes were recorded outside the E protein. It is important to note that in comparison of SA14 to SA14-14-2, the E-E179K substitution is dictated by a single nucleotide reside change, G1117A, in the second base of the codon (AGC). However, in mouse brain G1117A is accompanied by an additional A1116G mutation in the first base of the codon such that the double mutation encodes the glutamine mutation (GAC). Due to incomplete viral sequence of SA14-2-8 virus in mouse brain by NGS, Sanger methods were used to show that no nucleotide changes took place in the E gene in the brains of AG129 mice.

Again, no consensus changes to the genome of SA14-2-8 were detected in the brains of A129 mice. As no mice infected with SA14-14-2 succumbed to infection, the brains were not assayed for viral titer as it was assumed that any infection would have been cleared by the end of the study (28 dpi).

## Discussion

In this manuscript, we have shown that, as expected, SA14-14-2 has the most favorable safety/immunogenicity profile of the SA14-derived vaccine strains in vivo. It is also clear that the mechanism of attenuation of SA14-14-2 is different to that of a previously investigated flavivirus LAV (YFV 17D), in that 17D is characterized by a highly homogenous genotype whereas that of SA14-14-2 is heterogenous. Interestingly, SA14-14-2 is the most diverse of the three vaccine strains derived from SA14. Nevertheless, both YF 17D and JE SA14-14-2 vaccines share a similar phenotype in mouse models, good immunogenicity, cross-protective response and impressive safety profile making them highly effective LAVs^55^.

SA14-14-2 vaccine is produced in PHK cells using a seed-lot system much like that of 17D though the YFV vaccine is produced in chicken eggs^56^. In this substrate, SA14-14-2 has been shown that primary, master, working and commercial seeds (up to eight passages) and an experimental 17 passages of the virus maintain a stable genotype^9^. When these genomic sequences are compared to other SA14-14-2 GenBank submissions, it is clear that consensus sequence changes occur with extended passage in PHK cells or passage outside of this cell substrate (Table 1)^7,57^. This was shown in these studies where passage of SA14-14-2 three times in Vero cells and one in C6/36 cells to generate the working stock resulted in two additional nucleotide consensus changes, one a non-synonymous change at nucleotide 1512 (E-K179E) and the other a synonymous change at nucleotide 9122 (NS5-482G). As E-179E is the consensus residue in SA14-2-8 it would suggest that either a lysine or glutamine can be present at this residue without affecting attenuation in humans.

The differences between the published genomic sequences of SA14-14-2^7–12^ are explained by the apparently high levels of genetic diversity of this vaccine strain. This may be considered a concern for a LAV but the phenotypic data reported in this paper are very similar to that of the commercial vaccine indicating that the attenuated phenotype is maintained despite the apparent genetic variation. In fact, the mean diversity level of SA14-14-2 is only slightly, though statistically significantly, lower than WT parent strain SA14. As SA14-14-2 is highly susceptible to the antiviral Ribavirin, the cause of genetic plasticity could be localized to the NS proteins. Resistance to Ribavirin, a GTP analog, has been shown to be a marker of flavivirus replication complex fidelity^44^. Susceptibility to the drug suggests that the replication complex is not able to distinguish the analog from guanosine, which results in an increase in mutation frequency. The susceptibility of SA14-14-2 to Ribavirin differentiates the virus from yellow fever 17D, which was shown to be relatively resistant to Ribavirin^56^.

It is clear that the genetic diversity of SA14-14-2 does not make the virus susceptible to reversion to virulence. We hypothesize that this is because the genetic diversity of SA14-14-2 is not random upon passage, instead being localized to discrete regions yielding genotypes that retain an attenuated phenotype. With this knowledge it can be assumed that previously submitted SA14-14-2 sequences are correct despite consensus changes, representing a selection for a different, attenuated consensus^8–12,50^. As the vaccine has an impressive clinical safety record, it can be assumed that this multigenic genotype is due to multiple attenuating residues that maintain the attenuated phenotype. This type of mechanism has been shown in other flavivirus vaccine candidates and is the rationale for engineering attenuating mutations in multiple genes of rationally designed flavivirus vaccines^55,56^. In one such study, the multigenic nature of SA14-14-2 attenuation was shown using a chimeric vaccine, Chimerivax-JE, which utilizes SA14-14-2 prME genes in a YFV 17D backbone. Single reversions to WT did not have great effect on neurovirulence whereas groups of changes (especially clusters including E-F107L or E-K138E) did significantly increase virulence of the vaccine virus in mice. To this end, we have shown that the critical attenuating mutations at E-107 and E-138 are extremely stable in SA14-14-2 with no SNVs detected at these positions at any passage making it unlikely that these reversions would occur. This robust genotype may lend itself to the protective capacity of the vaccine, which has been shown to be affective against multiple JEV genotypes^8^.

The mechanism of SA14-14-2 diversity can be mapped using SA14-5-3, a progenitor of SA14-14-2, which is less genetically diverse and was over attenuated clinically. Only four amino acid residues in the replication complex (NS1-H351D, NS4B-H131D, NS5-H639Q and NS5-A671V) differentiate SA14-5-3 from SA14-14-2. Of these four residues, NS5-671 is potentially important as it extends into the pocket of the RdRp active site and is in close proximity to the conserved catalytic site in motif C of the RdRp (NS5-668). This site participates in binding GTP during pre-initiation of replication and NS5-671V is conserved in all other strains of JEV as well as WNV, YFV and DENV-2 suggesting that any alteration could affect the efficiency of viral replication and potentially susceptibility to ribavirin^58,59^. A more distantly related vaccine virus, SA14-2-8 was also overattenuated clinically but was resistant to Ribavirin, showing little loss of infectivity titer even at the highest concentration of drug tested. Interestingly, SA14-2-8 maintains an NS5 gene that is identical to WT SA14, which supports the hypothesis that the flavivirus replication complex as a whole, not exclusively the RdRp, contributes to viral diversity^60^. Furthermore, SA14-2-8 only differs from SA14 at four residues (NS1-P126S, NS2B-F7V, NS3-A105G and NS4A-I255V) in the replication complex, suggesting that these residues play a key role in restricting the viral diversity of SA14-2-8.

**Fig. 7:**
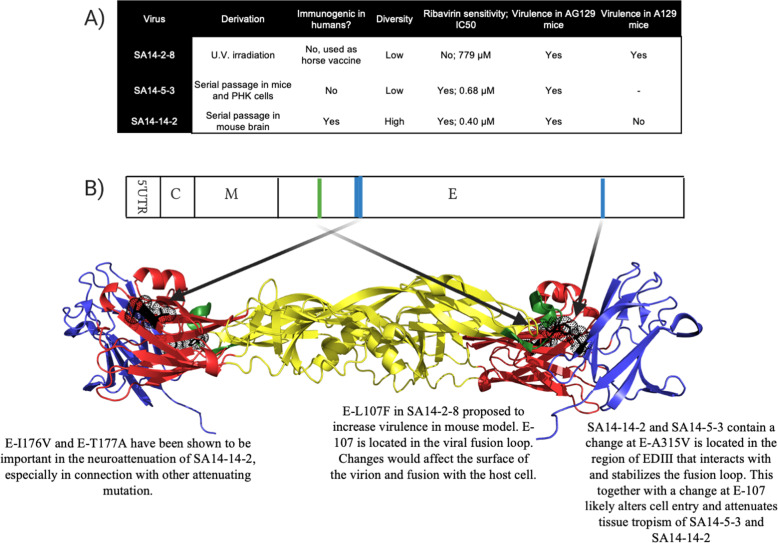
Summary of attenuating E protein mutations in JEV vaccine strains. The three attenuated vaccine strains were empirically derived which led to distinct combinations of genotypes and phenotypes (**A**). It is apparent that genetic diversity doesn’t correlate with attenuation in mice. It is proposed that changes to the E protein of SA14-2-8 (shown in green) and SA14-14-2 (shown in blue) are responsible for differences in mouse virulence (**B**).

Combining the results of the genotypic analysis and phenotypic profiles of the three vaccines, SA14-14-2 was the most genetically diverse of the vaccine strains and showed the most balanced safety/immunogenicity profile. In A129 mice, SA14-14-2 was completely attenuated, suggesting an important role for lack of type-2 interferon antagonism in the safety of the vaccine. SA14-5-3 and SA14-2-8 had very low levels of diversity and very few SNVs detected but radically distinct phenotypes in mice. SA14-5-3 was completely attenuated in the highly susceptible AG129 mouse model whereas SA14-2-8 was significantly more virulent than SA14-14-2 in both AG129 and A129 mouse models. As no reversions were detected in the brains of either AG129 or A129 mice, we speculate that this virulence phenotype is due to SA14-2-8 being more genetically similar to the parent strain WT SA14 than SA14-5-3. The two viruses differ by only 35 nucleotides and 13 amino acids, making SA14-2-8 more similar to its WT parent than to either of the two other vaccine strains tested. In particular, the maintenance of the WT residue at E-107 likely impacts the attenuation of SA14-2-8 in mice as it has been shown to be a major determinant of neurovirulence in SA14-14-2 (Fig. 7)^34^.

Although our studies have not identified a correlation to virulence, the genetic diversity differences between SA14-14-2 and SA14-2-8 could be important to vaccine immunogenicity as A129 mice that survived SA14-2-8 infection had significantly lower antibody titers compared to those infected with SA14-14-2. SA14-5-3 and SA14-14-2 are genetically very similar, with only three changes distinguishing the E proteins of the two vaccines. We postulate that the high frequency of SNVs in SA14-14-2 E and NS genes potentially affect the phenotype of the virus with respect to both immunogenicity and replicative efficiency. We also speculate that high frequency SNVs in the genotypic population of SA14-14-2 enabled the virus to cause disease in the highly susceptible AG129 model and caused the virus to be highly immunogenic in the A129 model. As SA14-14-2 contains a genetically diverse E gene (i.e., multiple SNVs at a high percentage of the RNA population) it is possible that the immune system sees a range of virions once administered, leading to a diversified immune response. In support of this hypothesis, WT strains of JEV show extensive genetic variation yet the SA14-14-2 vaccine is efficacious against all known strains of WT JEV^59,61,62^. Additionally, studies show that monoclonal antibodies induced by genotypes I and III viruses bind and neutralize viruses in other genotypes^63–65^. Our hypothesis is supported by the observation that these SNVs were not detected in the SA14-5-3 genome where the virus was 100% attenuated in the most susceptible model. In fact, the vaccine ampule passaged to create the working stock for these studies differed from the published vaccine sequence above (MH258852) at four nucleotides, which resulted in three amino acid substitutions (E-S47N, E-K279M, and NS4B-N131D)^10^. Interestingly, SNVs encoding the majority variant of the previously published commercial vaccine at each of these four positions were detected in the SA14-14-2 strain used here. This suggests that a subset of the viral population in the vaccine ampule used here was genetically identical to that of the previously published ampule^10^.

It should be emphasized that the presented findings do not extensively address the stability of the current vaccine genotype over time or passage, and are instead focused on genetic properties and phenotype of progenitor strains; the findings strongly suggest utility of using deep sequencing for monitoring of empiric vaccine population metastability to ensure both safety and protection characteristics.

The virulence of SA14-14-2 in both A129 and AG129 mouse models greatly resembled that of YFV 17D, which has an MST of 10.1 days in three to four week old AG129 mice by the subcutaneous route but is completely avirulent in age matched A129 inoculated by the same route^55^. Thus, our results are consistent in suggesting that NS genes and viral diversity have no role in attenuation of JE SA14-14-2 but possibly do play a role in immunogenicity and the E protein encodes determinants of attenuation, which is supported by amino acid substitutions involved in mouse virulence being located in the E protein (Fig. 7). Despite differing viral diversity, none of the vaccine strains display evidence of defective interfering RNA species by NGS, which was recently detected in the live attenuated influenza vaccine^66^. Thus, though 17D and SA14-14-2 maintain distinct genotypes, their phenotypes are comparable. The techniques employed during the generation of SA14-14-2 are likely responsible for its unique genotype and solid attenuated phenotype. SA14-5-3 and SA14-2-8 had been shown to be over attenuated in clinical trials leading vaccine developers to passage SA14-5-3 in suckling mice to increase the immunogenicity of the vaccine. This passaging likely selected a population for SA14-14-2 that was not clonal, unlike 17D which was passaged hundreds of times in chicken tissue. The diverse yet attenuated viral population of SA14-14-2 provides cocktail of antigens that induce robust and protective immune response and represents a unique attenuation mechanism for live-attenuated vaccines.

## Methods

### Passaging of viral stocks

A freeze-dried vaccine ampule of SA14-14-2 was obtained from NICPBP (Beijing) in 1987/1988. We do not have the lot number. Thus, the vaccine sample is derived from manufacture in the original production facility, which is different to the current vaccine that is manufactured in the new facility. The vaccine was reconstituted in HPLC grade water and passaged once in C6/36 and twice in Vero cells. SA14-2-8^12^ was passaged once in C6/36 cells and once in Vero cells. SA14-5-3^12^ was passaged three times in Vero cells. Wild-type (WT) strain SA14 was obtained from the World Reference Center for Emerging Viruses and Arboviruses (WRCEVA, Galveston, Texas). Passages in Vero and C6/36 cells occurred in minimal essential media (MEM) supplemented with L-glutamate, penicillin-streptomycin, non-essential amino acids, and 2% fetal bovine serum by volume. Viral infectivity assays were undertaken in Vero cells by focus forming assay using anti-JEV mouse immune ascites fluid kindly provided by the World Reference Center for Emerging Virus and Arboviruses (WRCEVA, UTMB), biotinylated goat anti-mouse IgG and NuetrAvidin-HRP (ThermoFischer)^71^.

### Next generation sequencing (NGS) of JEV vaccine strains

RNA was extracted from the seed stock and final passage of SA14 and SA14-14-2, plus the final two passages each of SA14-2-8 and SA14-5-3 using the Qiagen QIAmp viral RNA isolation kit (Qiagen). cDNA libraries were prepared using random hexamers (TruSeq RNA V2 kit) and sequenced on either the Illumina HiSeq1500 platform or the NextSeq550 platform by the UTMB NGS Core. Two viruses (SA14-14-2 and SA14-2-8) were sequenced on both platforms to evaluate consistency. No variability was identified. A de novo consensus sequence was generated for each sample using ABySS V1.3.7 assembly of paired end reads with k values from 20 to 40. The consensus sequences of each virus were then compared to previously published consensus sequences of the same strain using BioEdit (V 7.2.5).

The reads obtained from Illumina sequencing were trimmed to a minimum length of 35 bases and quality controlled (Q-score ≥30) using the open source software Trimmomatic (V 0.39). Reads were aligned using Bowtie2 (Version 2.2.6) very-sensitive-local presets to the de novo consensus sequences generated. The resulting alignment was sorted by coordinate using PicardTools (V 2.20.5) SortSam and PCR duplicates were removed using PicardTools MarkDuplicates. Shannon entropy was calculated using previously published means^44,60^. Single nucleotide variants (SNVs) were called using the open source software LoFreq V2 with a variant cutoff of 1%^67^. Shannon’s entropy and SNVs were calculated for all samples excluding the final passage of SA14-5-3, which had low coverage.

### Viral infectivity reduction of antiviral ribavirin

Viral infectivity reduction curves in the presence of ribavirin were undertaken as previously reported^44^. Briefly, Vero cells were inoculated with SA14, SA14-14-2, SA14-5-3 or SA14-2-8 at a multiplicity of infection of 0.05. Ribavirin was added at 2X concentration (1:2 dilution curve starting at 3 mM), allowed to incubate for 30 minutes at room temperature and then MEM supplemented with 2% FBS added to reduce the concentration to 1X. After 48 hours, cell culture supernatants were collected, and virus titrated via focus forming assay. Each assay was completed in triplicate.

### Mouse virulence studies

All animal procedures were approved by the University of Texas Medical Branch (UTMB) Institutional Animal Care and Use Committee and studies were carried out in compliance with the recommendations of the Guide for the Care and Use of Laboratory Animals (National Research Council).

AG129 (interferon α/β and *γ* receptor deficient) and A129 (interferon α/β receptor deficient) mice were bred and maintained at animal facilities at UTMB. Groups of six-week-old animals (*n* = 7–9) were inoculated subcutaneously (s.c.) with 1000 PFU of SA14-14-2, SA14-2-8 or SA14-5-3. For AG129 mouse experiments, two studies were undertaken with each virus and results combined for reporting. For A129 mice a single study was undertaken with SA14-14-2 and SA14-2-8.

Following inoculation mice were weighed daily and monitored for morbidity for 28 days. Animals exhibiting clinical signs of neurological disease or with weight loss >20% of initial body weight were humanely euthanized and counted as dead on the following day of the study for analysis. On day three post inoculation, blood was collected by retro-orbital bleed to quantify viremia using iScript cDNA synthesis kit (BioRad) and PF1S and PF2R-bis primers^68^. In addition, for some animals the brains were harvested at the time of euthanasia and homogenized using 1.5 mm zirconium beads. After clarification by centrifugation, viral load was determined using focus-forming assays and expressed as FFU/gram. Viral RNA was extracted using Qiagen Viral RNA isolation kits and sequenced using NGS. The E protein gene of viruses that returned incomplete sequences by NGS were Sanger sequenced using E protein primers described previously^69^ Only changes to the E protein are reported.

At the end of the study, surviving animals were sacrificed, serum was collected and heat-inactivated by incubation at 56 °C for 30 minutes. The samples were stored frozen at −20 °C until the neutralizing antibody titers were determined by focus reduction neutralization tests (FRNT_50_). Briefly, 2-fold serial dilutions were incubated with 50 FFU of infecting virus that was subsequently used to infect Vero cells. After four days, cells were fixed with acetone:methanol and plates stained as previously specified. Dilutions with a 50% reduction in infectivity titer were considered the FRNT_50_ value.

### Statistical analysis

All statistical analysis was competed using Prism 7 software (v7.0). A two-way ANOVA with Tukey’s multiple comparison test was used to compare the sensitivity of viruses to ribavirin. A four-parameter, non-linear regression was used to fit sensitivity curves and determine 50% inhibitory concentration (IC_50_). Ordinary, one-way ANOVA with Tukey’s multiple comparison tests were used to determine differences in mean Shannon entropy, viremia, FRNT_50_ and titer in the brain. Mantel-Cox tests were used to identify differences in survival curves.

### Reporting summary

Further information on research design is available in the Nature Research Reporting Summary linked to this article.

## Supplementary information

Supplementary Information

Reporting Summary

## Data Availability

All unique biological materials and the corresponding datasets generated and analyzed during the current study are available from the corresponding author on reasonable request. The genomes are available in Genbank (SA14 p0: MT764727; SA14-14-2 p0: MT764728; SA14-5-3: MT764729; SA14-2-8 p0: MT764730; SA14 p1: MT764731; SA14-14-2 p1: MT764732, and SA14-2-8 p1: MT764733). The NGS data are available in ArrayExpress (E-MTAB-9385)
