# Supplementary material for: Japanese encephalitis virus live attenuated vaccine strains display altered immunogenicity, virulence and genetic diversity
Source: NPJ Vaccines. 2021 Sep 2;6:112. doi: 10.1038/s41541-021-00371-y (PMC8413339; doi:10.1038/s41541-021-00371-y)
Supplement: Supplementary file 1 — Supplementary Information [file 41541_2021_371_MOESM1_ESM.pdf]

SUPPLEMENTARY INFORMATION

Supplementary Table 1: Comparison of SA14 in this study and six previously published genomic sequences in Genbank
Nucleotide position, coding sequence (CDS) nucleotide positions, polyprotein amino acid (AA) position, gene and amino acid position within protein are recorded. Nucleotide changes that do not occur within the CDS and therefore do not result in an amino acid change are recorded as (-). Nucleotide changes that are within the CDS but do not result in an amino acid change are shown as (*). Single letter amino acid residue codes are used below.

Nucleotide Position	CDS Position	Polyprotein	Gene	AA protein #	SA14	KU323483	U14163	KU821122	MH258848	KX254415	KU871316
538	443	148	prM	21	D	G					
1052	957	319	E	25	L		*				
1708	1613	538	E	244	G	E	E	E		E	E
2691	2596	866	NS1	72	R		*				
2843	2748	916	NS1	122	I		*				
3181	3086	1029	NS1	235	G		D		D	D	
3351	3256	1086	NS1	292	G		S				
4013	3918	1306	NS2A	97	P	*					
4427	4332	1444	NS2B	71	S			*			*
4519	4424	1475	NS2B	102	M		T				
4825	4730	1577	NS3	73	K		R		R		
5243	5148	1716	NS3	212	A		*				
5432	5337	1779	NS3	275	R			*			*
6051	5956	1986	NS3	482	D			N			N
6638	6543	2181	NS4A	58	V		*		*	*	
6700	6605	2202	NS4A	79	K		R				

Supplementary Table 2: Comparison of SA14-2-8 in this study and previously published genomic sequence of SA14-2-8 strain (U15763)

Nucleotide position, coding sequence (CDS) nucleotide positions, polyprotein amino acid (AA) position, gene and amino acid position within protein are recorded. Nucleotide changes that do not occur within the CDS and therefore do not result in an amino acid change are recorded as (-). Nucleotide changes that are within the CDS but do not result in an amino acid change are shown as (*). Single letter amino acid residue codes are used below.

Nucleotide Position	CDS Position	Polyprotein	Gene	AA protein #	SA14-2-8	U15763
739	644	215	prM	88	R	K
1354	1259	420	E	126	I	T
1360	1265	422	E	128	R	K
1456	1361	454	E	160	E	G
1921	1826	609	E	315	A	V
2408	2313	771	E	477	E	D
2441	2346	782	E	488	G	*
2691	2596	866	NS1	72	R	*
3085	2990	997	NS1	203	E	G
3284	3189	1063	NS1	269	D	*
3290	3195	1065	NS1	271	N	*
3535	3440	1147	NS1	353	F	S
3584	3489	1163	NS1	369	F	*
4233	4138	1380	NS2B	7	V	F
4825	4730	1577	NS3	73	K	R
4921	4826	1609	NS3	105	G	V
5396	5301	1767	NS3	263	H	*
6008	5913	1971	NS3	467	N	*
6035	5940	1980	NS3	476	G	*
6700	6605	2202	NS4A	79	K	R
6701	6606	2202	NS4A	79	K	*
6791	6696	2232	NS4A	109	L	*
7049	6954	2318	NS4A	195	G	*
7112	7017	2339	NS4A	216	T	*
7121	7026	2342	NS4A	219	A	*
7193	7098	2366	NS4A	243	T	*
7337	7242	2414	NS4B	24	A	*
7478	7383	2461	NS4B	71	N	*

7706	7611	2537	NS5	10	E	D
8099	8004	2668	NS5	141	D	*
8276	8181	2727	NS5	200	R	*
8394	8299	2767	NS5	240	L	*
8882	8787	2929	NS5	402	I	*
9603	9508	3170	NS5	643	K	E
9824	9729	3243	NS5	716	N	*
10211	10116	3372	NS5	845	G	*

Supplementary Table 3: Comparison of SA14-5-3 in this study and previously published genomic sequence in Genbank (U04521)

Nucleotide position, coding sequence (CDS) nucleotide positions, polyprotein amino acid (AA) position, gene and amino acid position within protein are recorded. Nucleotide changes that do not occur within the CDS and therefore do not result in an amino acid change are recorded as (-). Nucleotide changes that are within the CDS but do not result in an amino acid change are shown as (*). Single letter amino acid residue codes are used below.

Nucleotide Position	CDS Position	Polyprotein	Gene	AA protein #	SA14-5-3	U04521
20	-75	-25	5'UTR	-	N	K
576	481	161	prM	34	C	R
1708	1613	538	E	244	G	E

Supplementary Table 4: Comparison of SA14 to vaccine strains used in this study.

Nucleotide position, coding sequence (CDS) nucleotide positions, polyprotein amino acid (AA) position, gene and amino acid position within protein are recorded. Nucleotide changes that do not occur within the CDS and therefore do not result in an amino acid change are recorded as (-). Nucleotide changes that are within the CDS but do not result in an amino acid change are shown as (*). Single letter amino acid residue codes are used below.

					SA14		SA14-14-2		SA14-5-3		SA14-2-8	
Nucleotide Position	CDS Position	Protein Position	Gene	Codon within Gene	Nucleotide	Amino Acid	Nucleotide	Amino Acid	Nucleotide	Amino Acid	Nucleotide	Amino Acid
39	-56	-18	5'UTR	#N/A	T	-	A	-	A	-	-	-
292	197	66	C	66	T	L	C	S	C	S	-	-
739	644	215	prM	88	A	K	-	-	-	-	G	R
1061	966	322	E	28	T	D	C	*	C	*	C	*
1117	1022	341	E	47	A	N	G	S	-	-	-	-
1296	1201	401	E	107	C	L	T	F	T	F	-	-
1389	1294	432	E	138	G	E	A	K	A	K	A	K
1456	1361	454	E	160	G	G	-	-	-	-	A	E
1503	1408	470	E	176	A	I	G	V	G	V	G	V
1506	1411	471	E	177	A	T	G	A	-	-	-	-
1512	1417	473	E	179	A	K	G	E	-	-	G	E
1661	1566	522	E	228	T	P	-	-	-	-	C	*
1769	1674	558	E	264	G	Q	T	H	-	-	-	-
1813	1718	573	E	279	A	K	-	-	T	M	-	-
1921	1826	609	E	315	C	A	T	V	T	V	-	-
2051	1956	652	E	358	C	N	-	-	-	-	T	*
2293	2198	733	E	439	A	K	G	R	G	R	G	R
2408	2313	771	E	477	C	D	-	-	-	-	A	E

2441	2346	782	E	488	G	G	A	*	-	-	-	-
2691	2596	866	NS1	72	C	R	A	*	A	*	-	-
2843	2748	916	NS1	122	T	I	C	*	C	*	-	-
3181	3086	1029	NS1	235	G	G	A	D	A	D	A	D
3184	3089	1030	NS1	236	T	V	-	-	-	-	C	A
3351	3256	1086	NS1	292	G	G	A	S	A	S	-	-
3493	3398	1133	NS1	339	G	R	T	M	T	M	-	-
3528	3433	1145	NS1	351	G	D	C	H	-	-	-	-
3539	3444	1148	NS1	354	T	N	A	K	A	K	-	-
3599	3504	1168	NS1	374	G	E	A	*	A	*	A	*
3562	3467	1156	NS1	362	C	Q	T	L	T	L	-	-
3677	3582	1194	NS1	400	C	G	T	*	T	*	-	-
3776	3681	1227	NS2A	18	C	A	T	*	T	*	T	*
3801	3706	1236	NS2A	27	C	L	T	*	T	*	T	*
4106	4011	1337	NS2A	128	A	A	G	*	G	*	-	-
4233	4138	1380	NS2B	7	T	F	-	-	-	-	G	V
4402	4307	1436	NS2B	63	A	E	-	-	-	-	G	G
4403	4308	1436	NS2B	63	G	E	T	D	T	D	-	-
4408	4313	1438	NS2B	65	A	D	G	G	G	G	-	-
4782	4687	1563	NS3	59	A	M	G	V	G	V	-	-
4921	4826	1609	NS3	105	C	A	G	G	G	G	G	G
4922	4827	1609	NS3	105	T	A	C	*	C	*	C	*
5311	5216	1739	NS3	235	C	A	T	V	T	V	-	-
5396	5301	1767	NS3	263	C	H	-	-	-	-	T	*
6008	5913	1971	NS3	467	C	N	T	*	T	*	T	*
6035	5940	1980	NS3	476	G	G	-	-	-	-	A	*
6425	6330	2110	NS3	606	A	Q	G	Q	G	Q	-	-
6638	6543	2181	NS4A	58	T	V	C	*	C	*	C	*
6728	6633	2211	NS4A	88	G	T	A	*	-	-	-	-
6791	6696	2232	NS4A	109	G	L	-	-	-	-	C	*
6944	6849	2283	NS4A	160	A	A	G	*	-	-	-	-
7049	6954	2318	NS4A	195	G	G	-	-	-	-	T	*
7112	7017	2339	NS4A	216	A	T	-	-	-	-	T	*

7121	7026	2342	NS4A	219	C	A	T	*	T	*	T	*
7193	7098	2366	NS4A	243	C	T	T	*	T	*	T	*
7227	7132	2378	NS4A	255	A	I	G	V	G	V	G	V
7295	7200	2400	NS4B	10	A	G	T	*	-	-	-	-
7478	7383	2461	NS4B	71	C	N	-	-	-	-	T	*
7656	7561	2521	NS4B	131	G	D	A	N	-	-	-	-
7736	7641	2547	NS5	20	C	S	T	*	-	-	-	-
7751	7656	2552	NS5	25	T	F	-	-	-	-	C	*
8099	8004	2668	NS5	141	C	D	T	*	T	*	T	*
8276	8181	2727	NS5	200	C	R	T	*	T	*	-	-
8394	8299	2767	NS5	240	C	L	T	*	T	*	-	-
8832	8737	2913	NS5	386	C	H	T	Y	T	Y	-	-
8882	8787	2929	NS5	402	A	I	T	*	T	*	-	-
8891	8796	2932	NS5	405	C	V	T	*	T	*	T	*
9122	9027	3009	NS5	482	G	G	A	*	-	-	-	-
9593	9498	3166	NS5	639	G	Q	T	H	-	-	-	-
9688	9593	3198	NS5	671	T	V	C	A	-	-	-	-
9695	9600	3200	NS5	673	G	K	A	*	A	*	-	-
9818	9723	3241	NS5	714	C	C	T	*	T	*	-	-
10046	9951	3317	NS5	790	G	V	A	*	A	*	-	-
10139	10044	3348	NS5	821	C	V	T	*	T	*	-	-
10211	10116	3372	NS5	845	A	G	-	-	-	-	G	*
10217	10122	3374	NS5	847	T	R	C	*	C	*	-	-
10428	10333	3445	3'UTR	918	T	-	C	-	C	-	-	-
10950	10855	3619	3'UTR	1092	G	-	-	-	-	-	C	-
10951	10856	3619	3'UTR	1092	C	-	-	-	-	-	G	-

Supplementary Table 5: Consensus changes upon passage of SA14-14-2 (Panel A) and SA14-2-8 (Panel B)
Nucleotide position, coding sequence (CDS) nucleotide positions, polyprotein amino acid (AA) position, gene and amino acid position within protein are recorded. Nucleotide changes that do not occur within the CDS and therefore do not result in an amino acid change are recorded as (-). Nucleotide changes that are within the CDS but do not result in an amino acid change are shown as (*). Single letter amino acid residue codes are used below.

A)

Nucleotide Position	CDS Position	Polyprotein	Gene	AA protein #	SA14-14-2 Seed	SA14-14-2 C6/36	SA14-14-2 Vero p1	SA14-14-2 Vero p2
1512	1416	473	E	179	K			E
9122	9026	3009	NS5	482	G			G

B)

Nucleotide Position	CDS Position	Polyprotein	Gene	AA protein #	SA14-2-8 p3	SA14-2-8 p4
158	62	24	C	24	V	V
1447	1351	454	E	160	G	E
3482	3386	1132	NS1	338	V	V
4224	4128	1380	NS2B	7	F	V

Supplementary Table 6: Single Nucleotide Variants (SNVs) detected in final passage of SA14.
Nucleotide position, coding sequence (CDS) nucleotide positions, polyprotein amino acid (AA) position, gene and amino acid position within protein are recorded. Nucleotide changes that do not occur within the CDS and therefore do not result in an amino acid change are recorded as (-). Nucleotide changes that are within the CDS but do not result in an amino acid change are shown as (*). Single letter amino acid residue codes are used below.

Nucleotide Position	CDS Position	Consensus	Variant	Protein Position	Consensus	Variant	Gene	Codon within Gene	SNV Percentage
538	443	A	G	148	D	G	prM	21	1.59
613	518	A	G	173	E	G	prM	46	3.81
1025	930	T	C	310	S	S	E	16	4.10
1181	1086	C	T	362	I	I	E	68	7.97
1268	1173	T	C	391	T	T	E	97	1.50
1708	1613	G	A	538	G	E	E	244	21.02
2664	2569	C	T	857	L	L	NS1	63	4.78
2841	2746	A	G	916	I	V	NS1	122	1.68
3181	3086	G	A	1029	G	D	NS1	235	31.53
3473	3378	A	G	1126	G	G	NS1	332	4.57
4427	4332	C	T	1444	S	S	NS2B	71	18.01
4559	4464	C	T	1488	I	I	NS2B	115	3.66
4751	4656	A	G	1552	T	T	NS3	48	1.53
5432	5337	A	G	1779	R	R	NS3	275	14.72
6051	5956	G	A	1986	D	N	NS3	482	15.44
6638	6543	T	C	2181	V	V	NS4A	58	25.43
6911	6816	A	T	2272	A	A	NS4A	149	6.30
8190	8095	T	C	2699	L	L	NS5	172	3.34
8405	8310	A	G	2770	R	R	NS5	243	4.56
10413	10318	A	G	3440	-	-	3'UTR	-	2.11
10738	10643	C	G	3548	-	-	3'UTR	-	3.39
10745	10650	T	G	3550	-	-	3'UTR	-	3.45

Supplementary Table 7: Single Nucleotide variants (SNVs) detected in final passage of SA14-14-2.

Nucleotide position, coding sequence (CDS) nucleotide positions, polyprotein amino acid (AA) position, gene and amino acid position within protein are recorded. Nucleotide changes that do not occur within the CDS and therefore do not result in an amino acid change are recorded as (-). Nucleotide changes that are within the CDS but do not result in an amino acid change are shown as (*). Single letter amino acid residue codes are used below.

Nucleotide Position	CDS Position	Consensus	Variant	Protein Position	Consensus	Variant	Gene	Codon within Gene	SNV Percentage
134	39	T	C	13	A	A	C	13	2.62
572	477	C	T	159	N	N	prM	32	4.10
1116	1021	A	G	341	S	G	E	47	37.01
1117	1022	G	A	341	S	N	E	47	33.10
1241	1146	T	C	382	S	S	E	88	24.46
1383	1288	A	G	430	K	E	E	136	1.29
1453	1358	T	A	453	V	D	E	159	1.93
1512	1417	G	A	473	E	K	E	179	38.14
1891	1796	C	T	599	T	I	E	305	2.80
1911	1816	A	G	606	K	E	E	312	4.93
2120	2025	C	T	675	S	S	E	381	1.08
2198	2103	T	C	701	F	F	E	407	4.54
2411	2316	A	T	772	R	R	E	478	3.20
2558	2463	C	T	821	A	A	NS1	27	1.28
3068	2973	C	T	991	D	D	NS1	197	2.12
3113	3018	T	C	1006	L	L	NS1	212	1.41
3458	3363	T	C	1121	N	N	NS1	327	1.13
3652	3557	T	G	1186	V	G	NS1	392	1.27
3669	3574	C	T	1192	L	F	NS1	398	5.70
3695	3600	G	C	1200	L	F	NS1	406	2.09
3845	3750	C	T	1250	N	N	NS2A	41	22.71
4181	4086	C	T	1362	A	A	NS2A	153	4.63
4294	4199	A	G	1400	E	G	NS2B	27	1.95
4367	4272	T	C	1424	D	D	NS2B	51	1.40

5261	5166	A	G	1722	R	R	NS3	218	1.01
5311	5216	T	C	1739	V	A	NS3	235	1.20
5582	5487	T	C	1829	P	P	NS3	325	5.84
5699	5604	G	A	1868	A	A	NS3	364	1.42
5835	5740	G	A	1914	D	N	NS3	410	1.97
7167	7072	G	A	2358	V	M	NS4A	235	2.97
7475	7380	C	T	2460	V	V	NS4B	70	2.88
7652	7557	C	T	2519	N	N	NS4B	129	1.74
7656	7561	A	G	2521	N	D	NS4B	131	17.40
7657	7562	A	G	2521	N	S	NS4B	131	11.84
7774	7679	T	C	2560	I	T	NS5	33	3.03
7862	7767	C	A	2589	L	L	NS5	62	1.20
8791	8696	C	T	2899	A	V	NS5	372	1.89
9050	8955	A	G	2985	G	G	NS5	458	2.65
9122	9027	G	A	3009	G	G	NS5	482	38.60
9788	9693	C	A	3231	G	G	NS5	704	26.12
10329	10234	G	A	3412	V	I	NS5	885	1.52
10419	10324	C	T	3442	-	-	3'UTR	-	5.68
10536	10441	A	C	3481	-	-	3'UTR	-	1.04
10696	10601	T	C	3534	-	-	3'UTR	-	7.59
10738	10643	C	G	3548	-	-	3'UTR	-	3.14
10745	10650	T	G	3550	-	-	3'UTR	-	2.67
10823	10728	G	T	3576	-	-	3'UTR	-	1.06

Supplementary Table 8: Single Nucleotide variants (SNVs) detected in final passage of SA14-2-8.
Nucleotide position, coding sequence (CDS) nucleotide positions, polyprotein amino acid (AA) position, gene and amino acid position within protein are recorded. Nucleotide changes that do not occur within the CDS and therefore do not result in an amino acid change are recorded as (-). Nucleotide changes that are within the CDS but do not result in an amino acid change are shown as (*). Single letter amino acid residue codes are used below.

Nucleotide Position	CDS Position	Consensus	Variant	Protein Position	Consensus	Variant	Gene	Codon within Gene	SNV Percentage
1456	1361	A	G	454	E	G	E	160	10.29
4233	4138	G	T	1380	V	F	NS2B	7	1.46
5797	5702	A	G	1901	K	R	NS3	397	1.13
10738	10643	C	G	3548	-	-	3'UTR	-	4.15
10745	10650	T	G	3550	-	-	3'UTR	-	3.88

Supplementary Table 9: Single Nucleotide Variants (SNVs) detected in final passage of SA14-5-3.
Nucleotide position, coding sequence (CDS) nucleotide positions, polyprotein amino acid (AA) position, gene and amino acid position within protein are recorded. Nucleotide changes that do not occur within the CDS and therefore do not result in an amino acid change are recorded as (-). Nucleotide changes that are within the CDS but do not result in an amino acid change are recorded as (*). Single letter amino acid residue codes are used below.

Nucleotide Position	CDS Position	Consensus	Variant	Protein Position	Consensus	Variant	Gene	Codon within Gene	SNV Percentage
26	-69	G	T	-23	-	-	5'UTR	-	1.22
38	-57	T	C	-19	-	-	5'UTR	-	5.06
1296	1201	T	C	401	F	L	E	107	1.38
1632	1537	C	T	513	H	Y	E	219	2.41
2869	2774	C	T	925	S	F	NS1	131	1.80
4908	4813	G	C	1605	E	Q	NS3	101	1.18
5111	5016	T	A	1672	G	*	NS3	168	4.57
5311	5216	T	C	1739	V	A	NS3	235	20.38
5672	5577	T	C	1859	Y	*	NS3	355	3.46
5835	5740	G	A	1914	D	N	NS3	410	1.37
5945	5850	C	A	1950	N	K	NS3	446	40.55
7055	6960	C	T	2320	S	*	NS4A	197	3.28
7167	7072	G	A	2358	V	M	NS4A	235	1.47
9370	9275	T	G	3092	M	R	NS5	565	1.52
9413	9318	G	A	3106	V	*	NS5	579	5.65
10211	10116	A	G	3372	G	*	NS5	845	1.00
10426	10331	A	G	3444	-	-	3'UTR	-	8.54
10428	10333	C	T	3445	-	-	3'UTR	-	1.21
