# Supplementary material for: Japanese encephalitis virus live attenuated vaccine strains display altered immunogenicity, virulence and genetic diversity
Source: NPJ Vaccines. 2021 Sep 2;6:112. doi: 10.1038/s41541-021-00371-y (PMC8413339; doi:10.1038/s41541-021-00371-y)
Supplement: Supplementary file 2 — Reporting Summary [file 41541_2021_371_MOESM2_ESM.pdf]

nature research

Corresponding author(s): Alan Barrett, PhD

Last updated by author(s): 08/01/2020

Reporting Summary

Nature Research wishes to improve the reproducibility of the work that we publish. This form provides structure for consistency and transparency in reporting. For further information on Nature Research policies, see our Editorial Policies and the Editorial Policy Checklist.

Statistics

For all statistical analyses, confirm that the following items are present in the figure legend, table legend, main text, or Methods section.

n/a	Confirmed	
☐	☒	The exact sample size (*n*) for each experimental group/condition, given as a discrete number and unit of measurement
☐	☒	A statement on whether measurements were taken from distinct samples or whether the same sample was measured repeatedly
☐	☒	The statistical test(s) used AND whether they are one- or two-sided<br>*Only common tests should be described solely by name; describe more complex techniques in the Methods section.*
☐	☒	A description of all covariates tested
☐	☒	A description of any assumptions or corrections, such as tests of normality and adjustment for multiple comparisons
☐	☒	A full description of the statistical parameters including central tendency (e.g. means) or other basic estimates (e.g. regression coefficient) AND variation (e.g. standard deviation) or associated estimates of uncertainty (e.g. confidence intervals)
☐	☒	For null hypothesis testing, the test statistic (e.g. *F*, *t*, *r*) with confidence intervals, effect sizes, degrees of freedom and *P* value noted<br>*Give P values as exact values whenever suitable.*
☒	☐	For Bayesian analysis, information on the choice of priors and Markov chain Monte Carlo settings
☒	☐	For hierarchical and complex designs, identification of the appropriate level for tests and full reporting of outcomes
☐	☒	Estimates of effect sizes (e.g. Cohen's *d*, Pearson's *r*), indicating how they were calculated

Our web collection on statistics for biologists contains articles on many of the points above.

Software and code

Policy information about availability of computer code

Data collection	Sequencing data was collected using HiSeq 1500 and NextSeq 550 Illumina sequencing platforms.
Data analysis	A de novo consensus sequence was generated for each sample using ABySS V1.3.7 assembly of paired end reads with k values from 20 to 40. The consensus sequences of each virus were then compared to previously published consensus sequences of the same strain using BioEdit (V 7.2.5). The reads obtained from Illumina sequencing were trimmed to a minimum length of 35 bases and quality controlled (Q-score ≥30) using the open source software Trimmomatic (V 0.39). Reads were aligned using Bowtie2 (Version 2.2.6) very-sensitive-local presets to the de novo consensus sequences generated. The resulting alignment was sorted by coordinate using PicardTools (V 2.20.5) SortSam and PCR duplicates were removed using PicardTools MarkDuplicates. Shannon entropy was calculated using previously published means. Single nucleotide variants (SNVs) were called using the open source software LoFreq V2 with a variant cutoff of 1%.

For manuscripts utilizing custom algorithms or software that are central to the research but not yet described in published literature, software must be made available to editors and reviewers. We strongly encourage code deposition in a community repository (e.g. GitHub). See the Nature Research guidelines for submitting code & software for further information.

Data

Policy information about availability of data

All manuscripts must include a data availability statement. This statement should provide the following information, where applicable:

- Accession codes, unique identifiers, or web links for publicly available datasets
- A list of figures that have associated raw data
- A description of any restrictions on data availability

All unique biological materials and the corresponding datasets generated and analyzed during the current study are available from the corresponding author on reasonable request. GenBank accession numbers: SA14 seed: MT764727, SA14 passage: MT764731, SA14-14-2 seed: MT764728, SA14-14-2 passage: MT764732,

SA14-2-8 p1: MT764730, SA14-2-8 p2: MT764733 and SA14-5-3 p1: MT764729
ArrayExpress temporary accession number (released upon publication): E-MTAB-9385

Field-specific reporting

Please select the one below that is the best fit for your research. If you are not sure, read the appropriate sections before making your selection.

☒ Life sciences ☐ Behavioural & social sciences ☐ Ecological, evolutionary & environmental sciences

For a reference copy of the document with all sections, see nature.com/documents/nr-reporting-summary-flat.pdf

Life sciences study design

All studies must disclose on these points even when the disclosure is negative.

Sample size	Seven to nine mice were used per group in this study. This sample size was not determined before hand but was rather decided using knowledge of previous studies and the number of animals necessary to result in a well powered study.
Data exclusions	No data was excluded from analysis.
Replication	Antiviral studies were undertaken in triplicate.
Randomization	As inbred mice were utilized, randomization was not necessary as all animals had an identical genetic background.
Blinding	Blinding was not relevant to this study as infected and uninfected animals are required to be housed separately.

Reporting for specific materials, systems and methods

We require information from authors about some types of materials, experimental systems and methods used in many studies. Here, indicate whether each material, system or method listed is relevant to your study. If you are not sure if a list item applies to your research, read the appropriate section before selecting a response.

Materials & experimental systems

n/a	Involved in the study	
☒	☐	Antibodies
☐	☒	Eukaryotic cell lines
☒	☐	Palaeontology and archaeology
☐	☒	Animals and other organisms
☒	☐	Human research participants
☒	☐	Clinical data
☒	☐	Dual use research of concern

Methods

n/a	Involved in the study	
☒	☐	ChIP-seq
☒	☐	Flow cytometry
☒	☐	MRI-based neuroimaging

Eukaryotic cell lines

Policy information about cell lines

Cell line source(s)	Vero (ATCC® CCL-81)
Authentication	There was no additional cell line authentication beyond the ATCC authentication process
Mycoplasma contamination	The cell lines were tested for mycoplasma using ATCC mycoplasma kits and through the deep sequencing of whole cell RNA.
Commonly misidentified lines (See ICLAC register)	*Name any commonly misidentified cell lines used in the study and provide a rationale for their use.*

Animals and other organisms

Policy information about studies involving animals; ARRIVE guidelines recommended for reporting animal research

Laboratory animals	AG129 (6 week old, female) and A129 (6 week old, female) mice were bred and maintained at animal facilities at UTMB.
Wild animals	The study did not involve wild animals
Field-collected samples	The study did not involve field-collected samples.

Ethics oversight	All animal procedures were approved by the University of Texas Medical Branch (UTMB) Institutional Animal Care and Use Committee and studies were carried out in compliance with the recommendations of the Guide for the Care and Use of Laboratory Animals (National Research Council).

Note that full information on the approval of the study protocol must also be provided in the manuscript.
